# Navigated cup implantation in hip arthroplasty: A meta-analysis

**DOI:** 10.3109/17453670903350073

**Published:** 2009-10-01

**Authors:** Johannes Beckmann, Dirk Stengel, Markus Tingart, Jürgen Götz, Joachim Grifka, Christian Lüring

**Affiliations:** ^1^Department of Orthopedic Surgery, University of RegensburgBad AbbachGermany; ^2^Depts f Trauma and Orthopaedic Surgery, Unfallkrankenhaus Berlin and University of GreifswaldGermany

## Abstract

**Background and purpose** Many studies have suggested that navigation-based implantation can improve cup positioning in total hip arthroplasty (THA). We conducted a systematic review and meta-analysis to compile the best available evidence, and to overcome potential shortcomings because of small sample sizes in individual studies.

**Methods** The search strategy covered the major medical databases from January 1976 through August 2007, as well as various publishers' databases. The internal validity of individual studies was evaluated independently by 3 reviewers. We used random-effects modeling to obtain mean differences in cup angulation and relative risk (RR) of cup positioning outside Lewinnek's safe zone.

**Results** Of 363 citations originally identified, 5 trials of moderate methodology enrolling a total of 400 patients were included in the analysis. Mean cup inclination and anteversion were not statistically significantly different between the conventional groups and the navigated groups. Navigation reduced the variability in cup positioning and the risk of placing the acetabular component beyond the safe zone (RR = 0.21, CI: 0.13–0.32).

**Interpretation** Based on the current literature, navigation is a reliable tool to optimize cup placement, and to minimize outliers. However, long-term outcomes and cost utility analyses are needed before conclusive statements can be drawn about the value of routine navigation in THA.

## Introduction

The work flow in operating rooms worldwide has been markedly influenced by computer-assisted surgery (CAS) (Stindel et al. 2007). About 10 years after its introduction, many applications are available for orthopedic and trauma procedures (Jenny 2006, Holly and Foley 2007, Stindel et al. 2007). CAS has gained acceptance, especially for arthroplasty of the knee and hip (Amiot and Poulin 2004, Stindel et al. 2007, Bauwens et al. 2007). There are 3 types of imaging systems used to simultaneously generate different planes of the target object, all of which need intraoperative registration of anatomical landmarks (Sikorski and Chauhan 2003). Either CT-based, fluoroscopically-assisted, or imageless methods are used to simultaneously generate different planes of the therapeutic object to be treated (Grutzner et al. 2004, Widmer and Grutzner 2004, Ottersbach and Haaker 2005, Honl et al. 2006, Kalteis et al. 2006a).

Recent studies have shown that even experienced surgeons often fail to place the acetabular component within Lewinnek's “safe zone” (i.e. inclination of 40° ± 10°, anteversion of 15° ± 10°) (Lewinnek et al. 1978) when using a freehand technique (Saxler et al. 2004a, Tannast et al. 2005a, Honl et al. 2006, Kalteis et al. 2006a, Bosker et al. 2007, Leichtle et al. 2007).

On the other hand, preliminary results from laboratory studies, larger case series, and multicenter experience suggest that navigation-based implantation improves cup positioning in THA (Saxler et al. 2004b, Honl et al. 2006, Minoda et al. 2006, Kalteis et al. 2006a, Leichtle et al. 2007, Parratte and Argenson 2007, Sugano et al. 2007). However, conflicting statements and suspected methodological limitations in an arbitrary sample of the studies that we reviewed led us to conduct a systematic review of the international literature on navigated THA with emphasis on cup orientation.

We wanted to compile the current best evidence by pooling all RCT and quasi-RCT studies of comparisons between navigated and conventional cup positioning in THA, and to examine whether they support the assumption of better radiographic and clinical results with navigation.

## Methods

We identified all investigations that (1) compared navigation-based THA and conventional THA with emphasis on cup implantation, regardless of the underlying condition, disease, or navigation system (ITT), and that (2) met a level of evidence of II or higher, according to the suggestions of the Oxford Center for Evidence-Based Medicine (i.e. prospective cohort study, low-quality RCT, quasi-RCT, and individual RCT). We made no restrictions about language.

Study designs representing a lower level of evidence, especially retrospective cohort studies, were excluded from the analysis. We reasoned that only experimental and quasi-experimental designs minimize the risk of confounding, and allow valid estimates of the efficacy of navigation.

Our search strategy covered all major medical databases (Medline, Embase, SciSearch, Cinahl, and the Cochrane Central Register of Trials) from January 1976 through August 2007.

We used the following medical subject headings, or their equivalents: ‘position*’, ‘orient*’, ‘inclin*’, ‘anteversion’, ‘dislocation’, ‘luxation’, ‘wear’, ‘loosening’, ‘computer assisted’, ‘computer based’, ‘imageless’, ‘image based’, ‘CT-based’, ‘navig*’, ‘CAOS’, ‘CAS’, each in combination with ‘hip’, ‘cup’, ‘arthroplasty’, ‘THA’ ‘prospective’, ‘meta’, ‘review’ and ‘random*’. We also scanned publishers’ databases and conducted manual searches in the Journal of Bone and Joint Surgery (American and British Volumes, including supplements), Clinical Orthopaedics and Related Research, Journal of Arthroplasty, and Acta Orthopaedica. The bibliographies of the papers identified were searched for additional relevant citations. Potentially eligible studies were selected by taking the title and abstract. If the title and the abstract were inadequate to reach a final decision, we obtained the full paper.

The internal validity of individual studies was evaluated independently by 3 reviewers (JB, CL, and DS). We assessed the following methodological issues: (1) Did the authors put forward a clear study hypothesis? (2) Did they perform a sample-size calculation? (3) Did they report their results according to the CONSORT statement (including an illustration of the flow)? (4) did they respect the intention-to-treat principle (e.g. were patients who had been assigned to navigated THA still analyzed as navigated if the system had failed? (5) Did they provide sufficient numerical information in order to be able to recalculate the results reported?

To test the hypothesis that cup placement in THA is more precise with navigation (compared to the conventional technique), we focused on the inclination and anteversion of the cup as target criteria. We also used criteria according to Lewinnek's ‘safe zone’ to investigate this hypothesis.

### Statistics

We abstracted and tabulated baseline details of patients enrolled in individual studies, where available (e.g. age, sex, underlying condition). Weighted means and weighted mean differences in inclination and anteversion between navigated and conventional cup placement were calculated with their 95% confidence intervals (CIs). We also computed the risk ratio (RR) of cup placement outside Lewinnek's ‘safe zone’. Heterogeneity was assessed with chi-square statistics. A p-value of < 0.1 was considered suggestive of statistical heterogeneity, prompting random effects modeling.

We attempted to measure publication bias—that is, a lack of small studies without significant results—by the linear regression test for funnel plot asymmetry described by Egger et al. (1997). However, because of the small sample of eligible studies, this was meaningless. Also, the sample size prohibited random-effects meta-regression to adjust common effect estimates for potential confounders.

All analyses were performed in an exploratory fashion. We used the STATA statistical software package version 10.0 (StataCorp, College Station, TX) for all analyses.

## Results

### Search results

**Figure 1.**
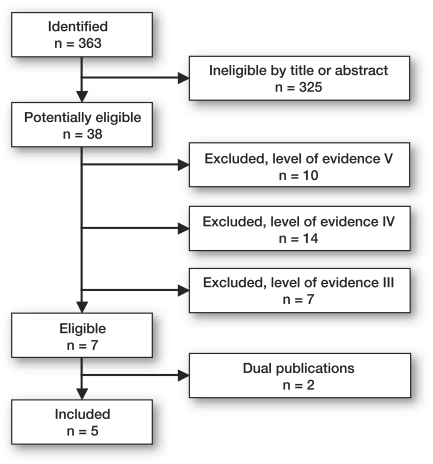
Study selection process according to QUOROM (Quality of Reporting of Meta-Analyses) standards.

Our search strategy revealed 363 citations, 326 of which were excluded after scanning the title and the abstract. 37 clinical reports were considered potentially eligible for this meta-analysis and were retrieved as full text. The study flow according to the QUOROM (Quality of Reporting Meta-Analyses) is depicted in Figure 1. Identified and excluded studies are listed in Tables 1 and 3 (See Appendix).

**Table 1.** Demographic baseline data

Author	Year	Conventional				Navigation			
		n	Mean age, years (SD)	No. of male patients	Primary OA	n	Mean age, years (SD)	No. of male patients	Primary OA
Leenders	2002	50	65 (–)	21	38	50	61 (–)	21	40
Stipcak	2004	25	57 (8)	13	20	25	54 (11)	19	20
Ottersbach	2005	50	60 (12)	22	–	50	59 (13)	27	–
Kalteis	2006a	30	65 (9)	13	30	60	64 (9)	30	60
Paratte	2007	30	63 (10)	16	26	30	61 (13)	16	27
– : not specified.									

**Table 2.** Studies included in the meta-analysis, with details of methodology

Author	Year	Cup	Navigation system	IRB approval	Clear hypothesis	Sample size calculation	Randomization procedure	ITT analysis	CONSORT flow diagram
Kalteis	2006	Press-fit (Pinnacle, DePuy, Warsaw, IN)	VectorVision hip 3.0 system (BrainLAB, Heimstetten, Germany)	yes	yes	yes	“by lot”	no	no
Paratte	2007	Press-fit (Hilock, Symbios, Yverdon, Switzerland)	Praxim Medivision, Grenoble, France	yes	yes	–	Indexed as RCT; actually matched pair design	no	no
Stipcak	2004	Press-fit (Plasma- cup, Aesculap, Nemêcko, Czech Republic)	OrthoPilot (B. Braun Aesculap)	–	yes	–	–	no	no
Ottersbach	2005	Press-fit Plasma-cup (n = 91), cemented PE (n = 9)	OrthoPilot (B. Braun Aesculap)	–	–	–	“by random ” no principle	no	
Leenders	2002	Uncemented, metal-backed cup	Surgi-Gate, Medivision, Oberdorf, Switzerland	–	yes	–	Indexed as RCT; actually mixed cohort study and RCT	no	no
– : not specified; IRB: institutional review board; ITT: intention-to-treat; CONSORT: Consolidated Standards of Reporting Trials; RCT: randomized controlled trial.									

The selection procedure left 5 eligible studies involving 400 enrolled patients (198 men, 202 women) with a mean age of 61 (SD 25) years. Of these, 2 studies were published in English, 2 in German, and 1 was published in the Czech language. 4 studies specified the underlying etiology of the osteoarthritis (OA), with 261/300 replacements (87%) performed because of primary OA. Patient samples were well balanced with regard to the basic demographic items available (Table 1).

One trial (Parratte and Argenson 2007) was published twice, in French and English. We included only the English paper. The authors’ line, IRB reference number, recruitment period, and number of subjects noted in another paper was suggestive of continued work (Kalteis et al. 2005, Kalteis et al. 2006a). We only included the most recent study in our analysis, which was a three-arm trial (CT-based navigation versus imageless-navigation versus conventional cup positioning). Since both navigation methods showed similar trends compared to conventional surgery—proportion of cups outside the safe zone: CT-based 5/30 (0.2, CI: 0.1–0.4), imageless 2/30 (0.1, CI: 0.1 – 0.2), freehand 16/30 [0.5, CI: 0.3–0.7)—results of the computer-assisted procedures were merged to facilitate analysis and to increase power.

Altogether, the methodological quality was moderate (Table 2). 1 trial indexed as RCT was, in fact, a matched-pair analysis in which “the first patient was randomly chosen and then one patient was selected out of every eight patients on a list of all patients meeting the inclusion criteria who were candidates for a THA. The patients assigned to the freehand cup placement group were matched for gender, age within five years, pathological condition, operatively treated side, and body-mass index within 3 points.” (Leenders et al. 2002). They mixed a cohort design with an RCT. The authors reported on 50 patients undergoing THA at their department prior to the establishment of a navigation system. Another 100 patients were randomly allocated to either CAS or conventional surgery. Of note, while the precision in cup positioning improved over time, there was no difference between navigated and freehand cup placement in the RCT part of the study. We only included the results from randomly assigned patients. The reasoning for the target sample size was reported in a single paper (Kalteis et al. 2006a). None fulfilled the ITT principle or represented a consort flow diagram. Studies provided no detailed information on complication rates, length of hospital stay, functional scoring, and other clinically relevant outcomes, or on costs or cost utility.

### Treatment results

**Figure 2.**
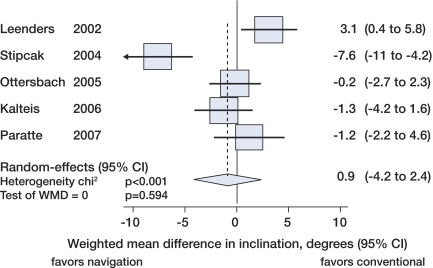
Forest plot showing that there was no statistically significant difference in mean inclination of cups placed with and without navigational support. Mean effect sizes of individual studies are expressed as squares, with larger squares denoting larger sample sizes, higher precision, and higher relative weight within the meta-analysis. Values lower than zero favor navigation and values higher than zero favor conventional cup positioning. The diamond shows the pooled overall effect size with the 95% confidence interval. When the 95% confidence interval includes the zero, it can be assumed that there is no statistical significance at the two-tailed p < 0.05 level.

**Figure 3.**
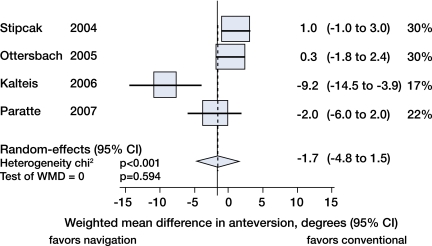
Forest plot showing that there was no statistically significant difference in mean anteversion of cups placed with and without navigational support. No information on anteversion was available in the trial by Leenders et al. (Leenders et al. 2002).

Cup inclination averaged 44° (CI: 40 – 48) in the conventional arm and 43° (CI: 40 – 46) in the navigation arm. The weighted mean difference in inclination between conventional and computer-assisted positioning was not statistically significant (–0.89°, CI: -4.2–2.4) (Figure 2). Means from Leenders' trial had to be derived from a histogram. When excluding this trial from random-effects pooling, the mean difference between groups was –0.30° (CI: -0.83–0.22). Cup anteversion averaged 17° (CI: 11–22) in the conventional arm and 15° (CI: 11–18) in the navigation arm. Again, this difference was compatible with chance (Figure 3).

**Figure 4.**
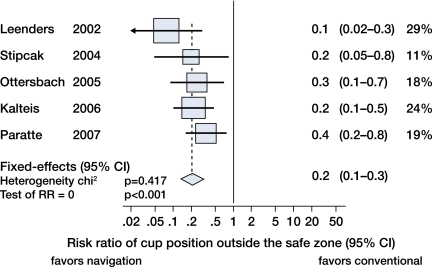
Forest plot showing the statistically significantly reduced relative risk of cup positioning outside the safe zone with navigation.

Overall, navigation reduced the variability in cup positioning statistically significantly, and reduced the risk of placing the acetabular component beyond the safe zone (Figure 4). The pooled RR of 0.21 (CI: 0.13–0.32) translates to a risk difference of 37% (CI: 45–29) in favor of navigation.

## Discussion

Correct cup positioning is crucial for the short- and long-term success of THA. Many studies have suggested that there is improved cup positioning with navigation-based implantation (Saxler et al. 2004a, Honl et al. 2006, Kalteis et al. 2006a, Leichtle et al. 2007, Parratte and Argenson 2007). However, individual studies are too small to allow conclusive statements on the potential benefit of navigation in THA.

Our meta-analysis demonstrates a clear advantage of navigated cup orientation over conventional freehand cup orientation in THA. As discussed later, however, various severe pitfalls and possible inherent error or bias must be considered. As with total knee arthroplasty and screw positioning in spinal surgery, the major benefit of navigation is the reduction of outliers, that is, cup positioning beyond the “safe zone” with an inclination of 40° (± 10°) and anteversion of 15° (± 10°) (Saxler et al. 2004a, Honl et al. 2006, Kalteis et al. 2006a, Minoda et al. 2006, Leichtle et al. 2007, Parratte and Argenson 2007, Sugano et al. 2007). Moreover, it seems that navigation-based cup positioning in THA meets the criteria of evidence by reducing the amount of outliers in cup orientation (Leenders et al. 2002, Stipcak et al. 2004, Ottersbach and Haaker 2005, Kalteis et al. 2006a, Parratte and Argenson 2007).

The findings from experimental and quasi-experimental investigations are supported by those from observational studies that were excluded from the present meta-analysis. Sugano et al. (2007) found none of 59 navigated cups as compared to 31 of 111 conventional implanted cups to be outside the “safe zone” (p < 0.001). There was no significant difference in mean inclination, but a significantly greater mean anteversion with conventional cup placement (p < 0.001). In a multicenter study, a significantly higher variability in both inclination and anteversion (p < 0.001) was found after conventional cup implantation (Saxler et al. 2004a).

In a minimally invasive THA study, significant variances in both inclination (p < 0.01) and anteversion (p < 0.03) were reported (Wixson and MacDonald 2005). In retrospective studies, a statistically significant difference in variation for both inclination and anteversion has been found (Haaker et al. 2007), and also an advantage in navigation-based cup placement in dysplastic hips (Haaker et al. 2003).

The reduction of outliers is of clinical relevance, as malpositioning of the acetabular component may cause impingement and restrict the range of motion. It is a known risk factor for dislocation and can lead to increased and premature wear, with elevated metal-ion concentrations in serum and an overall increased risk of loosening and revision (Patil et al. 2003, Brodner et al. 2004, Nishii et al. 2004).

The proven advantages of navigation must be traded off against the argument of prolonged surgery and higher costs (Eingartner 2007).

The number of studies, patients, and outcome data is still limited, and we also noted some weaknesses in trial methodology, which highlights various pitfalls and possible inherent error or bias that warrant further discussion. First, there was no clear evidence of publication error, and it is likely that the published information reflects the best results currently achievable with navigated cup positioning in THA. Future trials must adhere to methodological standards such as proper random assignment and intention-to-treat analyses, and aim for a thorough comparison of radiographic and functional results, complication and survival rates, quality of life, and also extra costs and cost utility.

Secondly, one uncertainty and limitation of evidence is the status of current discussion about the correct incorporation of the pelvic anatomy (Beckmann et al. 2008) regarding the generation of landmarks as a basis for imageless navigation (Lembeck et al. 2005, Richolt et al. 2005, Stiehl et al. 2005, Wolf et al. 2005, Mayr et al. 2006, Spencer et al. 2006, Beckmann et al. 2008) and the correct radiological assessment of the implant position (Olivecrona et al. 2004, Blendea et al. 2005, Tannast et al. 2005b, Jaramaz and Eckman 2006, Kalteis et al. 2006b, Liaw et al. 2006, Marx et al. 2006, Muller et al. 2006, Penney et al. 2007, Beckmann et al. 2008).

Thirdly, apart from cup orientation, outcomes such as longevity, range of motion, impingement, and dislocation further depend on the head-neck ratio, the offset, and the stem orientation (D'Lima et al. 2000, Widmer and Zurfluh 2004, Pedersen et al. 2005, Widmer and Majewski 2005, Masaoka et al. 2006, Yoshimine 2006, Malik et al. 2007, Widmer 2007). In addition, the surgical approach and endogenous factors such as comorbidity and muscular status may contribute to the fate of the hip joint (Soong et al. 2004, Zwartele et al. 2004, Meek et al. 2006).

Lastly, although we took care not to miss any relevant publication, we did not ask the authors for individual patient data or ongoing studies. Occasionally, editing of manuscripts and limited space in scientific journals may obscure some methodological features originally respected by study protocols.

In conclusion, based on the current literature, navigation is a reliable tool for optimization of cup placement in THA. Navigation reduces the incidence of outliers beyond the so-called desired “safe zone”. Long-term outcomes have to be awaited before making final statements about longevity of the prosthesis and patient satisfaction, which depend on factors other than just cup orientation. A corresponding cost utility analysis must also be done.

## APPENDIX

**Table 3.** Excluded studies

Author	Year	Journal	Level of evidence	Study description
Zheng	2002	Comput Aided Surg	V	mechanistic study, imageless navigation for cup positioning
Amiot	2004	Clin Orthop	V	cadaver study with repeated measurements of navigated cup positioning
Jolles	2004	Clin Orthop	V	mechanistic study, freehand vs. computer-assisted cup positioning
Kalteis	2004	Biomed Tech (Berl)	V	cadaver study of imageless navigation (VectorVision) for cup positioning
Nogler	2004	Clin Orthop	V	cadaver study, freehand vs. imageless navigation
Honl	2005	J Bone Joint Surg (Br)	V	mechanistic study of five different navigation systems (imageless and CT-based Navitrack, OrthoPilot, Surgetics Station, VectorVision) for cup positioning
Stiehl	2005	Comput Aided Surg	V	cadaver study, fluoroscopy-based navigated cup positioning
Tannast	2005	Comput Aided Surg	V	cadaver study, fluoroscopy-based navigated cup positioning
Belei	2007	Comput Aided Surg	V	cadaver study of navigated surface replacement
Cobb	2007	Clin Orthop	V	navigated cup positioning in sawbones
DiGoia	1998	Clin Orthop	IV	CT-based navigated cup positioning
Bernsmann	2001	Z Orthop Ihre Grenzgeb	IV	different cups and techniques influencing navigated cup positioning (Medivision, Optotrack)
DiGoia	2002	J Arthroplasty	IV	mechanical acetabular alignment guide for cup positioning
Hube	2003	Surg Technol Int	IV	CT-based and fluoroscopy-based systems for navigated cup positioning
Kiefer	2003	Int Orthop	IV	imageless navigation (OrthoPilot) for cup positioning
von Recum	2003	Unfallchirurg	IV	CT-free navigation (SurgiGate) for cup positioning
Wentzensen	2003	Int Orthop	IV	CT-free navigation (SurgiGate) for cup positioning
Grützner	2004	Injury	IV	imageless navigated cup postioning
Widmer	2004	Injury	IV	CT-based navigation for cup positioning
Dorr	2005	Iowa Orthop J	IV	imageless navigated cup postioning
Laffargue	2006	Rev Chir Orthop R A M	IV	imageless navigation for cup positioning
Blendea	2007	Comput Aided Surg	IV + V	cadaver and clinical studies of navigated cup positioning
Bosker	2007	Arch Orthop Trauma Surg	IV	freehand cup positioning - clinical estimation vs. radiological measurement
Dorr	2007	Clin Orthop	IV	clinical estimation vs. navigation accuracy, influence of the surgeon's experience on cup positioning
Haaker	2003	Z Orthop Ihre Grenzgeb	III	dysplastic hips, freehand vs. imageless navigation (SurgiGate) for cup positioning
Saxler	2004	Z Orthop Ihre Grenzgeb	III	freehand vs. imageless navigated (SurgiGate) cup positioning
Wixson	2005	J Arthroplasty	III	imageless navigated vs. freehand cup positioning
Saxler	2004	Int Orthop	III	freehand vs. imageless navigated (SurgiGate) cup positioning
Stipcak	2006	Acta Chir Orthop Traumatol Cech	III	freehand vs. imageless navigated (OrthoPilot) cup positioning with a minimally-invasive posterolateral approach
Haaker	2007	J Arthroplasty	III	retrospective, CT-based navigated vs. freehand cup positioning
Sugano	2007	J Bone Joint Surg (Br)	III	freehand vs. CT-based navigated cup positioning
